# Identification of lncRNAs involved in rice ovule development and female gametophyte abortion by genome-wide screening and functional analysis

**DOI:** 10.1186/s12864-019-5442-6

**Published:** 2019-01-28

**Authors:** Helian Liu, Ruihua Wang, Bigang Mao, Bingran Zhao, Jianbo Wang

**Affiliations:** 1 State Key Laboratory of Hybrid Rice, College of Life Sciences, Wuhan University, Wuhan, 430072 China; 2 State Key Laboratory of Hybrid Rice, Hunan Hybrid Rice Research Center, Changsha, 410125 China

**Keywords:** Rice, Long non-coding RNA, Ovule development, Female gametophyte abortion, Gene expression and regulation

## Abstract

**Background:**

As important female reproductive tissues, the rice (*Oryza sativa* L.) ovule and female gametophyte is significant in terms of their fertility. Long noncoding RNAs (lncRNAs) play important and wide-ranging roles in the growth and development of plants and have become a major research focus in recent years. Therefore, we explored the characterization and expression change of lncRNAs during ovule development and female gametophytic abortion.

**Results:**

In our study, whole-transcriptome strand-specific RNA sequencing (ssRNA-seq) was performed in the ovules of a high-frequency female-sterile rice line (*fsv1*) and a wild-type rice line (Gui99) at the megaspore mother cell meiosis stage (stage 1), functional megaspore mitosis stage (stage 2) and female gametophyte mature stage (stage 3). By comparing two rice lines, we identified 152, 233, and 197 differentially expressed lncRNAs at the three ovule developmental stages. Functional analysis of the coherent target genes of these differentially expressed lncRNAs indicated that many lncRNAs participate in multiple pathways such as hormone and cellular metabolism and signal transduction. Moreover, there were many differentially expressed lncRNAs acting as the precursors of some miRNAs that are involved in the development of ovules and female gametophytes. In addition, we have found that lncRNAs can act as decoys, competing with mRNAs for binding to miRNAs to maintain the normal expression of genes related to ovule and female gametophyte development.

**Conclusion:**

These results provide important clues for elucidating the female gametophyte abortion mechanism in rice. This study also expands our understanding about the biological functions of lncRNAs and the annotation of the rice genome.

**Electronic supplementary material:**

The online version of this article (10.1186/s12864-019-5442-6) contains supplementary material, which is available to authorized users.

## Background

Long noncoding RNAs (lncRNAs) are defined as RNAs whose lengths are greater than 200 bp and that lack protein-encoding function. According to the relative positions of lncRNAs in the genome and adjacent protein coding genes, they can be divided into five categories: (1) sense lncRNAs; (2) antisense transcripts (NATs) derived from introns; (3) intergenic noncoding (nc) RNAs (lincRNAs); (4) intronic nc RNAs (incRNAs); and (5) bidirectional lncRNAs [1]. Based on their mechanism of action, the function of lncRNAs can be grouped into four classes: signal, decoy, guide, and scaffold [2]. The lncRNAs can act as signal molecules for regulating gene transcriptional activity, and the spatial, temporal and expression status of regulatory factors can be judged by the expression of lncRNAs [3]. As decoy molecules, lncRNAs can directly or indirectly achieve the regulation of the expression of the target genes by recruiting some RNA-binding proteins [4]. The third mode of action of lncRNAs is that they direct the ribonucleoprotein complex to localize at a particular site [5]. In addition to the above three mechanisms, lncRNAs can also serve as scaffold, and their specific domain can bind to different types of proteins or transcription factors to form a skeleton complex, thereby regulating the effector elements upstream or downstream of the genes to activate or inhibit gene transcription [6]. Previous studies have shown that lncRNAs can recruit chromatin remodeling complexes to specific genomic sites where the chromatin is then modified to cause changes in gene expression [7, 8].

LncRNA-mediated regulation of coding genes at the transcriptional level can be divided into cis-regulation and trans-regulation. Cis-regulation refers to a lncRNA derived from a gene promoter or intergenic region that combines with a transcription factor to activate or inhibit the expression of an adjacent gene. For example, *FLOWERING LOCUS C* (*FLC*) is an important gene that regulates flowering time in plants, and its NAT lncRNA, COOLAIR, regulates the *FLC* gene through cis-regulation and thus affects flowering time [9, 10]. Respondingly, trans-regulation involves lncRNAs that bind to transcription factors or alter a transcription factor’s subcellular localization to activate or inhibit gene expression at another site, or they assist in the conversion of a protein from an inactive to an active state to regulate downstream gene expression. The lncRNA COLDAIR transcribed from the *FLC* intron region silences the *FLC* gene by binding to the PcG protein complex and inhibiting the methylation of *FLC-*associated histones [10]. In addition, some studies have found that lncRNAs can be transcribed from the antisense strands of genes, and such lncRNAs are often involved in the posttranslational splicing, editing, transport, translation and degradation of mRNA. LncRNAs not only participate in the formation of small RNAs but also act as trans-regulatory factors to regulate the formation of other small RNAs, and some lncRNAs can directly bind to small RNAs to regulate the functions of miRNAs [11].

In recent years, there has been a growing amount of evidence that suggests that lncRNAs are involved in the sexual reproduction of plants [12, 13]. Aberrant expression of lncRNAs can lead to defects in or abortion of gametophyte development. For example, in Arabidopsis, a natural antisense lncRNA, *asHSFB2a,* affects female gametophyte development by controlling the heat-stress transcription factor HSFB2a, and the development of female gametophytes is impaired when *asHSFB2a* expression levels are abnormal [14]. In addition, the long-day-specific male-fertility-associated RNA (LDMAR), a lncRNA of 1236 bases, regulates photoperiod-sensitive male sterility in rice. Under long-day conditions, the reduced transcription of LDMAR leads to premature programmed cell death in anther development and causes male sterility [12].

As rice is an important cereal crop and monocotyledonous model plant, clarifying its reproductive mechanism has important theoretical significance and practical value. The ovule is a female reproductive organ in rice that plays important roles in the process of reproduction. In ovules, megaspore mother cells (MMCs) undergo meiosis to form functional megaspores, and then, the functional megaspores form female gametophyte structures through three rounds of mitosis. The development of the female gametophyte and ovule sporophytic tissue are carried out simultaneously, and the development of the female gametophyte is completely dependent on the ovule sporophytic tissue for nutrient transport and mechanical support. In recent years, researchers have identified crosstalk between the ovule sporophytic tissue and female gametophyte [15]. Changes in gene expression in the ovule sporophytic tissue will affect the normal growth and development of the female gametophyte, and gene expression in the female gametophyte will also affect ovule sporophytic tissue [16–19].

A high-frequency female-sterile mutant rice line (*fsv1*) was used in our experiment. Phenotypic analysis showed abnormal development of female gametophytes in *fsv1*, in which most of functional megaspores degraded in advance and could not undergo normal mitosis [18]. Furthermore, the miRNA profiles of *fsv1* and Gui99 ovule development and female gametophytic abortion have been identified by high-throughput sequencing [20]. These findings will help us further reveal the mechanism of female gametophytic abortion and guide the present study.

In this study, high-throughput sequencing methods were used to analyze lncRNAs and their target genes during ovule development in *fsv1* and Gui99. The purpose of this work was to elucidate the expression profiles and regulatory mechanisms of lncRNA during ovule development and female gametophyte abortion. The study will provide some clues for further elucidation of ovule development and female gametophyte abortion.

## Methods

### Plant materials

Two lines of rice (*Oryza sative* ssp. *indica*), a high-frequency female-sterile rice line (*fsv1*) and a rice wild-type line (Gui99) were used for these experiments. The *fsv1* line is a genetically stable mutant obtained by introducing the genomic DNA of *Panicum maximum* into rice (cultivar Gui99) via ear-stem injection. Approximately 80.5% of the female gametophytes were aborted in *fsv1*, and other traits were similar to Gui99, including pollen fertility and plant morphology. The seeds of *fsv1* and Gui99 were obtained from State Key Laboratory of Hybrid Rice, Hunan Hybrid Rice Research Center, Changsha, China, and provided by Dr. Bingran Zhao and Dr. Bigang Mao. A detailed description of *fsv1* can be found in [18, 21]. The material plants were grown and maintained in the greenhouse of Wuhan University, Wuhan, China. Based on the known correspondence between the morphological characteristics of rice florets and the developmental period of the female gametophyte [22], we collected ovules in the MMC meiosis stage (stage 1), functional megaspore mitosis stage (stage 2) and female gametophyte mature stage (stage 3). The ovules were removed from the ovary with needles under stereomicroscopy, then rapidly placed in liquid nitrogen for RNA extraction. For each sample, we randomly selected 200 spikelets from 50 plants in the growing season, and performed three biologic replicates for each sample.

### RNA extraction, ss-RNA library construction and sequencing

Total RNA was extracted with Trizol (Invitrogen, Burlington, ON, Canada) according to the manufacturer’s instructions. The concentration and purity of the RNA and the OD_260_ / OD_280_ ratio were determined using an Agilent 2100 bioanalyzer. The ribosomal RNA in the total RNA was removed using the Ribo-Zero™ rRNA removal kit, and then the RNA was fragmented at random. Using the fragmented RNA as templates, the first strand of cDNA was synthesized by reverse transcription with a random six-base primer. After that, the cDNA secondary chain was synthesized by replacing dTTP with dUTP, followed by purification, end repair, and the addition of A bases and adaptors. For PCR amplification, the Illumina-specific Taq enzyme did not reach the U-base of the secondary chain of the cDNA, while the first strand of the cDNA was amplified. The libraries were subjected to quality control and quantitative PCR analysis. Finally, the cDNA libraries were sequenced by Illumina HiSeq4000, and the sequencing length was 100 bp.

### Identification of lncRNAs

After removing the adaptors and low quality reads of the raw data obtained from Illumina sequencing, we evaluated the clean data. Using HISAT2 (Version 2.0.4), the clean reads were searched against the rice genome. The transcripts obtained were assembled using StringTie (Version 1.0.4) and then the final transcripts were generated with Cuffmerge. Furthermore, the transcripts that mapped to known genes or that contained no information were eliminated from further identification as lncRNAs. Using CPC (Coding Potential Calculator), CNCI (Coding Non Coding Index) and txCdsPredict to predict the coding potential of these sequences, requiring CPC and CNCI scores that were less than 0 and txCdsPredict scores less than 500 as indicators for potential lncRNAs. This analysis was combined with information from the Pfam protein database, ensuring that predicted lncRNA transcripts did not contain protein-coding domains. If at least three of the above four judgment methods were in agreement, the transcripts were determined to be lncRNAs.

### Differential expression analysis of lncRNAs

The HISTA2 software was used to align clean reads to the reference genome and then RSEM was performed to calculate the expression levels of genes and transcripts. To make gene expression data comparable across samples, it is necessary to standardize the gene expression levels. The standardized method used by RSEM (http://deweylab.biostat.wisc.edu/rsem) is FPKM. The FPKM-specific formula is as follows:

$$ \mathrm{FPKM}=\frac{10^6C}{NL/{10}^3} $$

Let FPKM (A) be the expression level of gene A, then C is the number of unique alignments to the gene A, N is the total number of haplotypes that align to the reference gene, and L is the number of bases in the gene A coding region. The FPKM method can eliminate the influence of gene length and sequencing difference on the calculation of gene expression levels, and the calculated gene expression level can be directly used for comparing differential gene expression among different samples.

DEGseq [23] software was used for the intergroup difference analysis. The false discovery rate (FDR) method was used to correct the *P* value in multiple tests [24]. Fold change ≥2.00 and FDR ≤ 0.001 were taken as thresholds to judge significant differences in lncRNA filter conditions.

### Prediction and functional analysis of target genes of differentially expressed lncRNAs

The functions of lncRNAs are executed on coding genes via cis- or trans-regulation, the lncRNA and its target coding genes were considered to be lncRNA-mRNA pairs. Correlation test was performed on the expression levels of lncRNAs and mRNAs in all samples. The statistical analysis method was as follows: calculating the Spearman and Pearson correlation coefficients [25] of lncRNAs and mRNAs, requiring Spearman values ≥0.6 and Pearson values ≥0.6. The basis for predicting cis- regulation is related to the positional relationship of lncRNA genes and coding genes on the genome. It was determined to be cis-regulatory if lncRNA gene were within 10 kb upstream or 20 kb downstream of coding genes. If there was an overlap between the lncRNA genes and coding genes, we made a detailed classification of the overlap, which helped us understand the details of cis-regulation [26, 27]. Since trans-regulation is not dependent on a positional relationship, RNAplex software [28] was used to analyze the binding energy of the lncRNA and coding genes according to sequence complementarity, and a binding energy of<-30 was determined to be a trans-regulatory system.

If there were differentially expressed target coding genes for lncRNAs, they were considered to be candidate genes of interest. To determine which functional modules these candidate coding genes were more focused on, we conducted GO functional analysis. The methods for the GO analysis were detailed in the description by Yang et al. [18].

### Prediction of miRNA precursor of lncRNA

Blast (http://blast.ncbi.nlm.nih.gov/Blast.cgi) was used to align lncRNAs to miRBase (http://www.mirbase.org) to find potential miRNA precursors, and any alignments between lncRNAs with miRNA precursors that were greater than 90% were selected.

### Analysis of lncRNA, miRNA and mRNA network interactions

lncRNAs target mRNAs through cis- or trans-actions. Coupled with the targeted relationship between miRNAs and mRNAs and the possible targeted relationships between miRNAs and lncRNAs, networks of lncRNA-miRNA-mRNA interactions were identified. The results of this analysis were visualized using Cytoscape (http://www.cytoscape.org) software. In the network diagram, the connections indicate possible regulatory relationships. The yellow square represents miRNAs, the blue square represents mRNAs, and the red square represents lncRNAs.

### Validation by real-time quantitative PCR

Seven lncRNAs and their target genes were randomly selected for verification of the ssRNA-Seq results by quantitative real-time PCR (qRT-PCR). Total RNA was extracted from ovules at different stages using Trizol (Invitrogen), and ovules at each stage from two rice lines were measured in three biological replicates. The mRNA was reverse-transcribed using oligo (dT) primers and SuperScriptIIReverse Transcriptase (Invitrogen). The primers were designed using Primer5 software for qRT-PCR and are listed in Additional file 1: Table S1. *OsActin1* [22] was taken as an internal reference control to standardized the results. The ABI Step One Plus Real-Time PCR System (Applied Biosystems) was used for qRT-PCR with the Thunderbird SYBR qPCR mix (Toyobo, Kita-ku, Osaka, Japan). The qRT-PCR amplification reactions were carried out via the following program: 95 °C for 10 min, 95 °C for 15 s and 72 °C for 20 s, the last two steps were performed for 40 cycles.

## Results

### Overview of the ssRNA library sequencing data

**Fig. 1:**
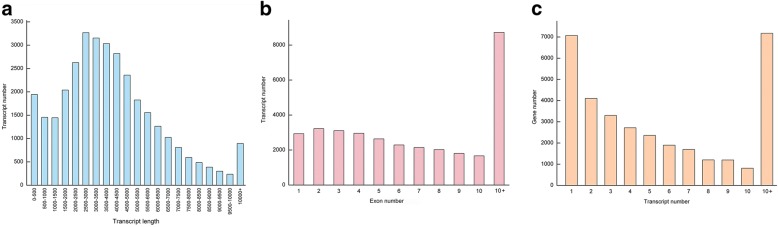
The qualitative analysis of assembled 33,518 novel transcripts. **a** Length distribution of novel transcripts; (**b**) Exon number distribution of novel transcripts; (**c**) The distribution of novel transcript number transcribed from genes

The samples of each stage were measured in three biological replicates. To elucidate the roles of lncRNAs in ovule development and female gametophyte abortion, a total of 18 ss-RNA libraries were constructed for deep sequencing on the Illumina 4000 platform. The Gui99 ovules at stages 1, 2 and 3 were represented by A1, A2 and A3; correspondingly, stage 1, 2 and 3 of *fsv1* ovules were represented by B1, B2 and B3, respectively. An average of 18 libraries with an output of approximately 12.74 Gb of data that has been submitted to the NCBI Sequence Read Archive (SRA) database was used (https://trace.ncbi.nlm.nih.gov/Traces/sra/sra.cgi?view=run_browser). The accession numbers of the 18 SRA runs are SRR8002616 - SRR8002633. HISAT2 was used to align clean reads to the rice reference genome and StringTie was used for assembly. Of the 18 library alignments, the uniquely mapping ratios between clean reads and the reference genome were 75.36–79.04%, suggesting that our sequencing results were reliable (Additional file 2: Table S2). After assembly, a total of 66,338 known transcripts were obtained. In addition, 33,518 novel transcripts were assembled, of which transcript lengths of 1500–5000 nt accounted for a high proportion, a large number of transcripts were found for more than ten exons, and a large number of genes were found having either one or more than ten transcripts (Fig. 1).

### Identification and quantitative analysis of lncRNAs

**Fig. 2:**
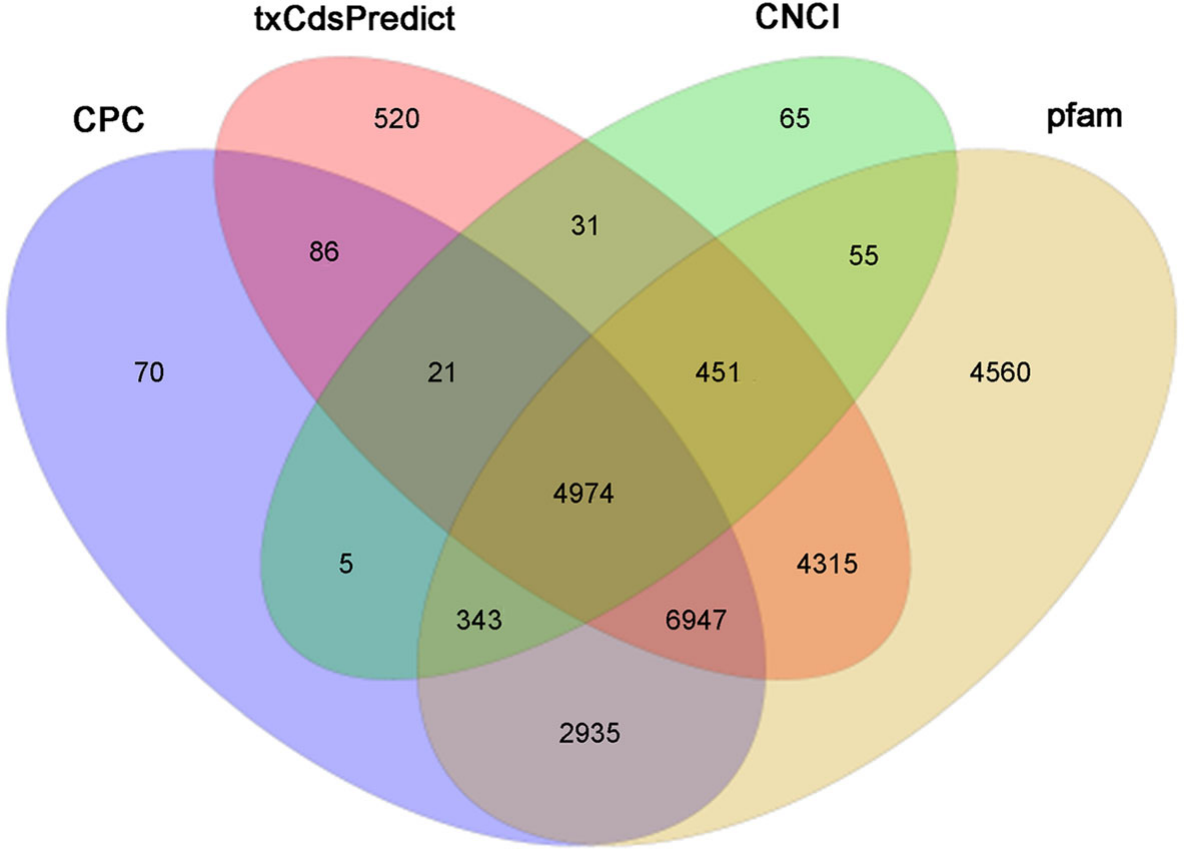
Three software CPC, txCdsPredict, CNCI and a protein database pfam were used to predict lncRNAs, and the transcript was determined when at least three of the four methods were consistent

**Fig. 3:**
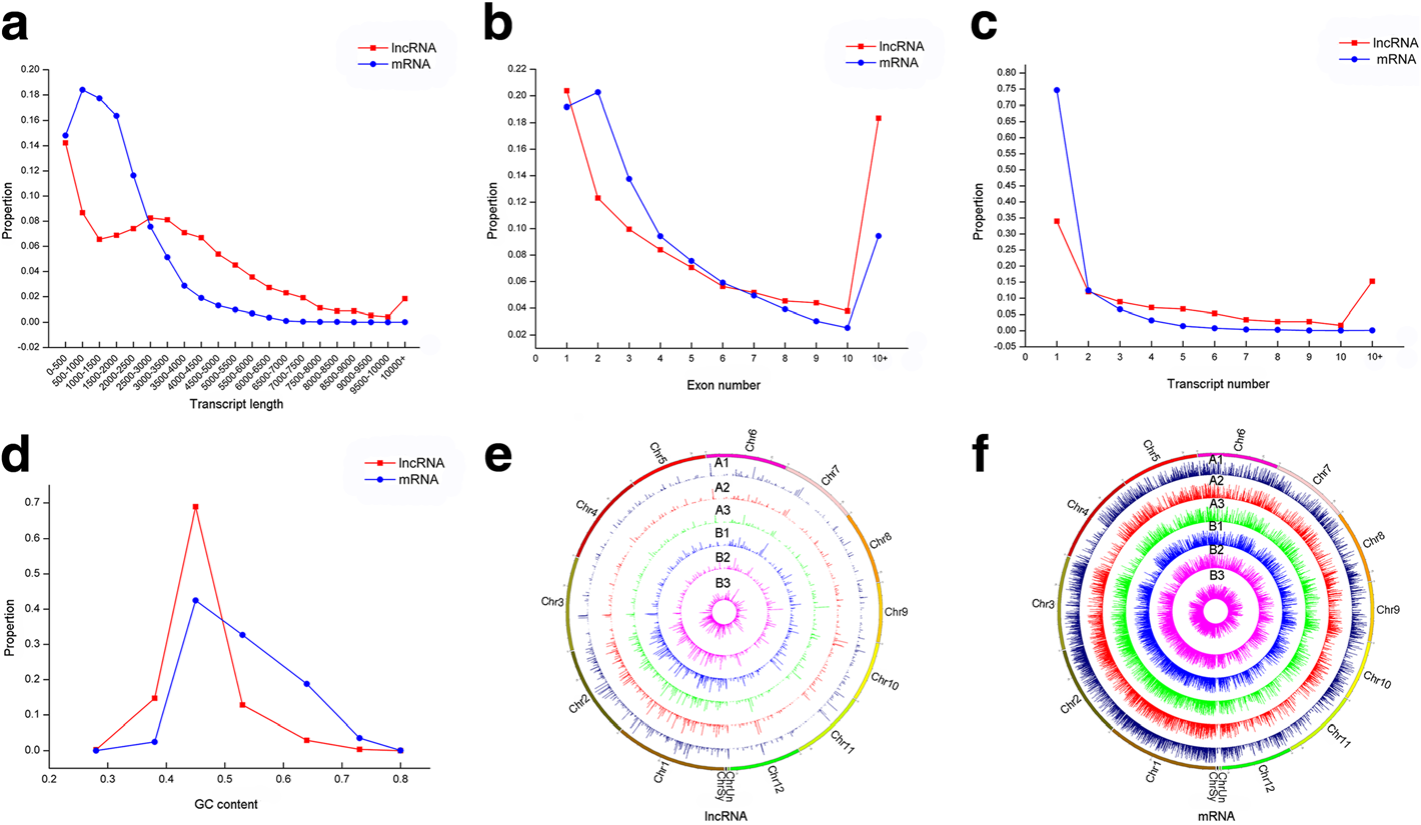
The comparative characteristics analysis of lncRNAs and mRNAs in ovules of Gui99 and *fsv1*. **a** The length distribution; (**b**) Exon number; (**c**) Transcript number; (**d**) GC content; (**e**) The expression level distribution of lncRNAs on 12 chromosomes; (**f**) The expression level distribution of mRNAs on 12 chromosomes

The CPC software, txCdsPredict, CNCI and the Pfam database were used to score the coding capacity of 33,518 novel transcripts simultaneously to identify lncRNAs. As a result, 12,736 novel transcripts were identified as lncRNAs (Fig. 2). Beyond that, 66,338 known transcripts were identified as mRNAs. The number of lncRNAs was very small compared to the number of coding RNAs, with 200–6500 nt transcripts representing 95% of the total number of lncRNAs. Most identified lncRNAs had only one exon in their transcripts, and most lncRNAs are derived from genes having only one transcript (Fig. 3a-c). Moreover, the GC content of lncRNAs varied from 23.61 to 79.25%, with an average content of 45.14%, while the GC content of mRNAs was relatively flat, with an average content of 52.88% (Fig. 3d).

**Fig. 4:**
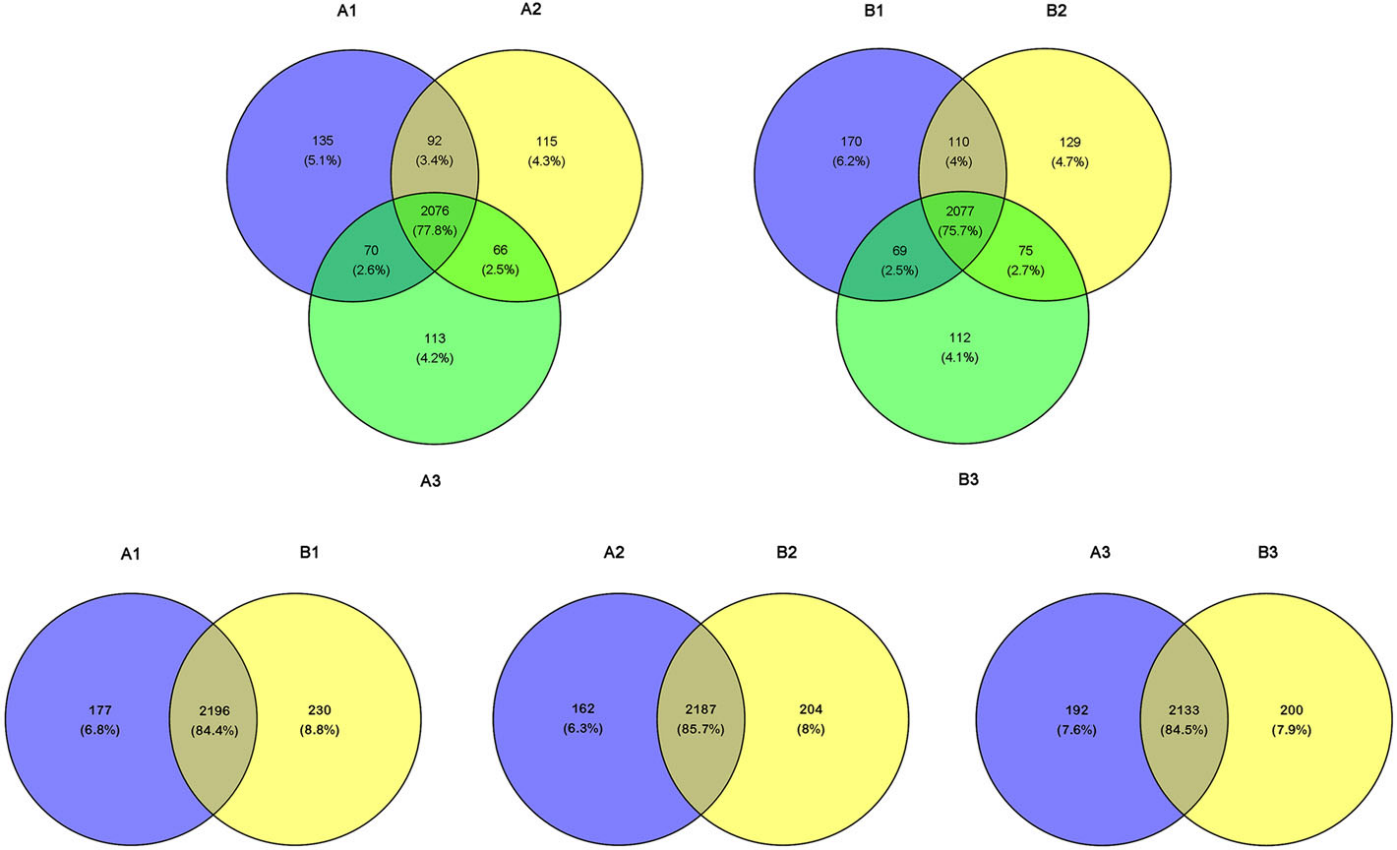
Venn diagram for novel lncRNAs expressed in Gui99 and *fsv1* ovules at three developmental stages. Total of 2076 lncRNAs were expressed at all three ovule developmental stages in Gui99, 92 lncRNAs were coexpressed in A1 and A2, 66 lncRNAs were coexpressed in A2 and A3, and 70 lncRNAs were coexpressed in A1 and A3. There were 135, 115 and 113 lncRNAs expressed exclusively in A1, A2 and A3, respectively. In *fsv1* ovules, 2077 lncRNAs were coexpressed in B1, B2 and B3. In addition, 110 lncRNAs were coexpressed in B1 and B2, 75 lncRNAs were coexpressed in B2 and B3, and 69 lncRNAs were coexpressed in B1 and B3. The number of exclusively expressed lncRNAs in B1, B2 and B3 were 170, 129 and 112, respectively. During ovule development, the number of coexpressed lncRNAs in Gui99 and *fsv1* were 2196, 2187 and 2133 at stage 1, stage 2 and stage 3, respectively. The number of exclusively expressed lncRNAs were 177 (A1) and 233 (B1) at stage 1, 162 (A2) and 204 (B2) at stage 2, and 192 (A3) and 200 (B3) at stage 3

RSEM was used to calculate the expression levels of genes and transcripts. In order to facilitate subsequent analysis during the three developmental stages in *fsv1* and Gui99 ovules, three biological replicates of each sample were homogenized and the final integration result was the expression level of lncRNA or mRNA (for example A1-A, A1-B, A1-C integrated into A1). The numbers of lncRNAs and mRNAs derived from expressed transcripts in each stage are shown in Table 1, and the normalized FPKM values for all lncRNAs and mRNAs in all samples were shown in Additional file 3: Table S3. Circos [29] software was used to visually describe the expression level distributions of lncRNAs and mRNAs on 12 chromosomes. The Circos results show that lncRNAs and mRNAs are highly expressed on chromosomes 1 and 2, and both the expression numbers and expression levels of mRNAs are higher than lncRNAs in the whole genome (Fig. 3e-f). In detail, 2965 lncRNAs (2373 lncRNAs in A1, 2349 lncRNAs in A2, 2325 lncRNAs in A3, 2426 lncRNAs in B1, 2391 lncRNAs in B2 and 2333 lncRNAs in B3) were obtained, and Venn diagrams were used to describe the expression of lncRNAs at three developmental stages in Gui99 and *fsv1* ovules (Fig. 4). During *fsv1* and Gui99 ovule development, most of the lncRNAs were continuously expressed, while some lncRNAs were only expressed at specific stages, suggesting that lncRNAs may contribute to the regulation of rice ovule development. In addition, the number of coexpressed lncRNAs decreased with the development of ovules in *fsv1* and Gui99 rice lines, indicating that lncRNAs may be involved in the regulation of female gametophyte fertility mainly at the early stage of ovule development.

**Table 1:** The number of lncRNAs and mRNAs derived from expressed transcripts in each ovule developmental stage of Gui99 and *fsv1*

Sample	LncRNA	mRNA
A1	2373	28,630
A2	2349	28,191
A3	2325	27,183
B1	2426	28,278
B2	2391	27,815
B3	2333	27,369

### Differentially expressed lncRNAs during the three developmental stages of *fsv1* and Gui99 ovules

**Fig. 5:**
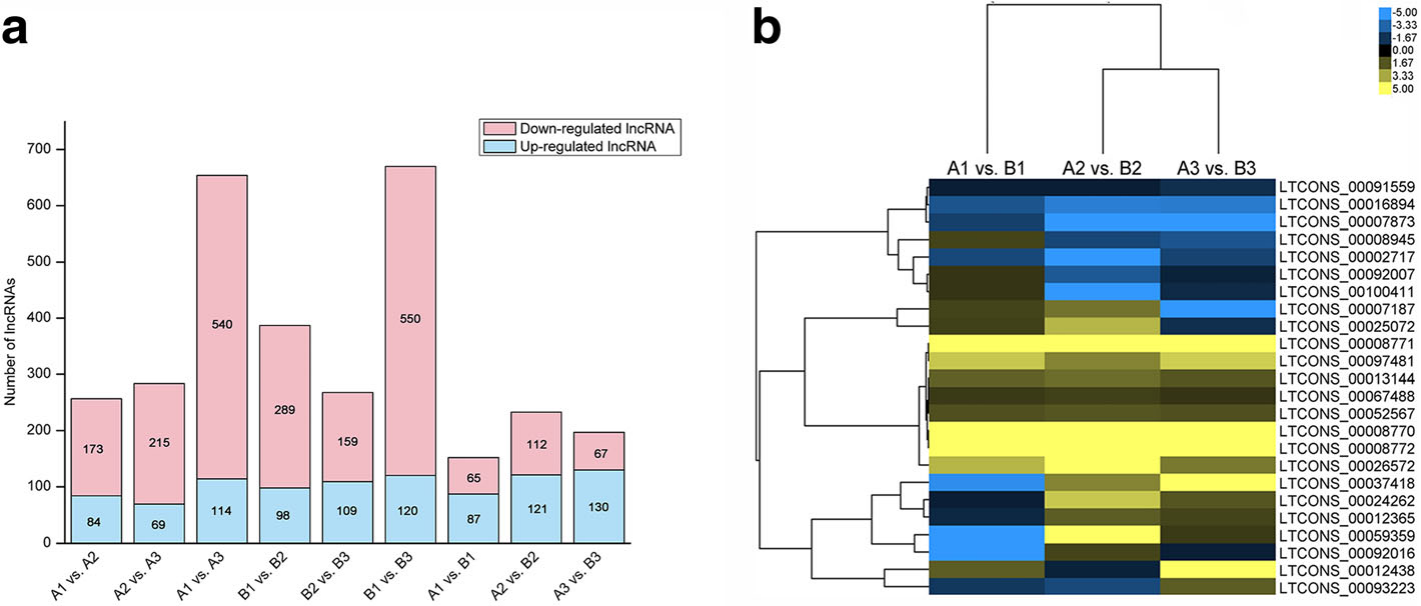
The profile of differentially expressed lncRNAs in *fsv1* and Gui99 ovules. **a** The distribution of upregulated and downregulated differentially expressed lncRNAs in each comparison. **b** Hierarchical cluster analysis of the differentially expressed lncRNAs in all three developmental stages. The color key represented FPKM normalized log_2_ transformed counts. The yellow color represented higher expression, and the blue color represented lower expression. Each column represented a comparison, while each row represented a lncRNA

The DEGseq software was used to calculate the expression levels of lncRNAs across different samples. The thresholds were ≥ 2.00 for fold change and ≤ 0.001 for FDR to define significant differential expression of lncRNAs. As a result, 257, 284, 654, 387, 268, 670, 152, 233 and 197 lncRNAs were found to be significantly differentially expressed in A1 vs. A2, A2 vs. A3, A1 vs. A3, B1 vs. B2, B2 vs. B3, B1 vs. B3, A1 vs. B1, A2 vs. B2 and A3 vs. B3, respectively (Additional file 4: Table S4). From stage 1 to stage 3, there were more downregulated lncRNAs than upregulated lncRNAs in Gui99 and *fsv1* ovules (Fig. 5a), indicating that downregulated lncRNAs occupied a larger proportion of differentially expressed lncRNAs during ovule development. To elucidate the role of lncRNA in rice female sterility, the lncRNA expression levels in *fsv1* and Gui99 were compared across ovules at the three developmental stages. There were 152, 233 and 197 lncRNAs that were significantly differentially expressed at stage 1, stage 2 and stage 3, respectively. In detail, 87 lncRNAs in *fsv1* ovules were upregulated and 65 were downregulated at stage 1, 121 upregulated and 112 downregulated lncRNAs at stage 2, and 130 upregulated and 67 downregulated lncRNAs at stage 3, compared with Gui99. Across the three stages of ovule development, the number of upregulated lncRNAs was always more than that of downregulated lncRNAs, suggesting that upregulated lncRNAs may play important roles in female gametophyte fertility. In addition, Cluster and JAVA TreeView software were used to cluster the lncRNAs that were differentially expressed in all three developmental stages of *fsv1* and Gui99 (Fig. 5b). The results showed that 8 of the 24 lncRNAs were continuously upregulated with ovule development and 4 were continuously downregulated, and these lncRNAs may be deeply involved in the process of female gametophytes formation.

### Prediction of coherent target protein-coding genes and functional analysis of lncRNAs

**Fig. 6:**
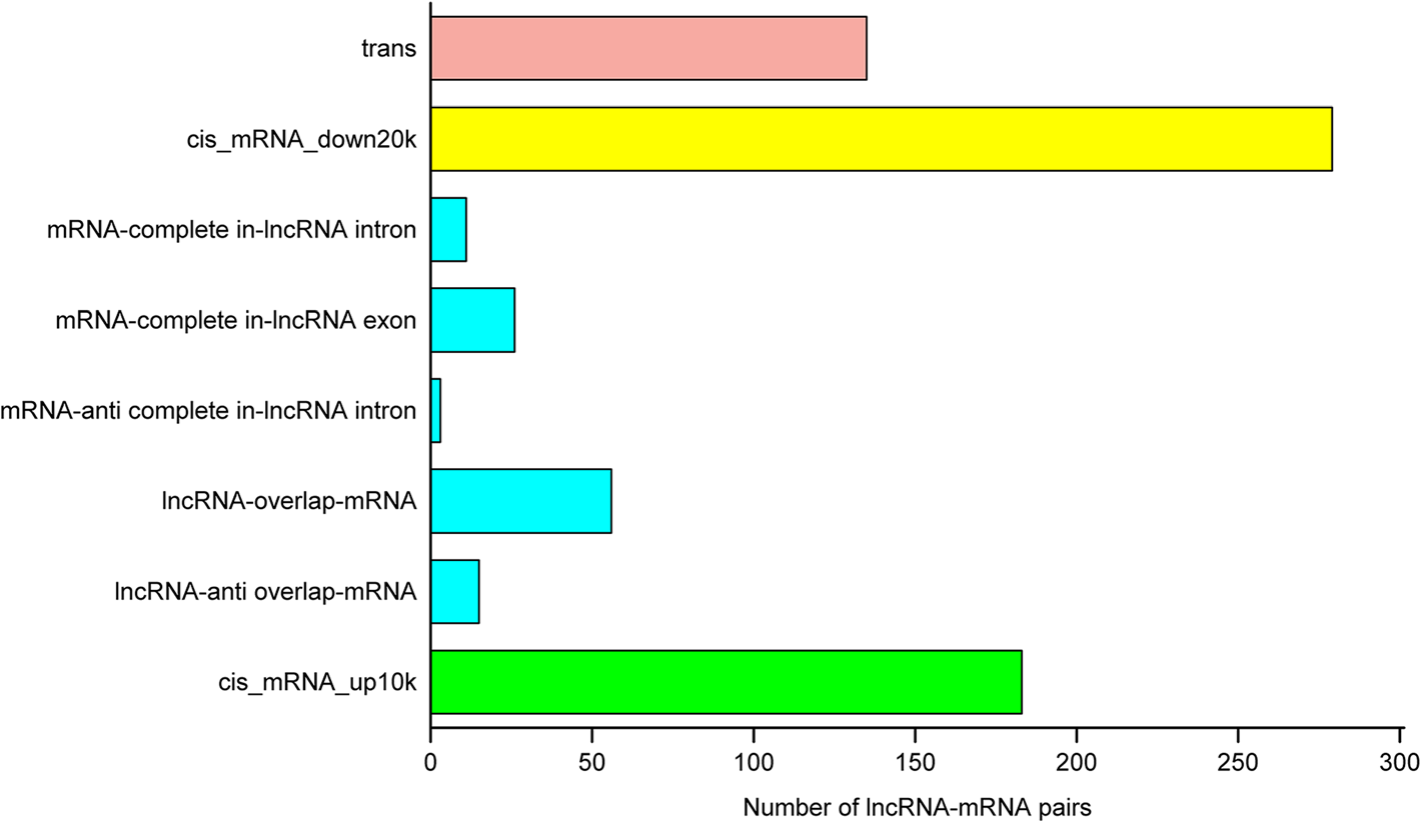
The number of different regulatory relationship types of lncRNA-mRNA pairs The green and yellow columns indicated that the target protein-coding genes of lncRNAs were located within 10 kb upstream and 20 kb downstream, and had no overlap with lncRNA. The blue column indicated the overlapping of lncRNAs and their target protein-coding genes. There are five types of overlapping: mRNA (coding gene) overlap with lncRNA transcript gene, mRNA anti-overlap with lncRNA, mRNA is completely located in the exon region or intron region of the lncRNA transcript gene, and the mRNA is completely located in the reverse intron of the lncRNA transcript gene. The All three below in the figure were cis-regulation. Besides, the red column was trans-regulation that calculated based on the binding energy of lncRNAs and mRNAs.

lncRNAs act via cis- and trans-regulation of target genes for biological function. As a result, a total of 708 matched lncRNA-mRNAs pairs for 353 differentially expressed lncRNAs and 579 mRNAs were predicted, of which 573 were cis-regulatory and 135 were trans-regulatory (Fig. 6 and Additional file 5: Table S5). From the data in Additional file 5: Table S5, it can be seen that there were positive and negative correlations between the expression of lncRNAs and their coherent target genes.

**Fig. 7:**
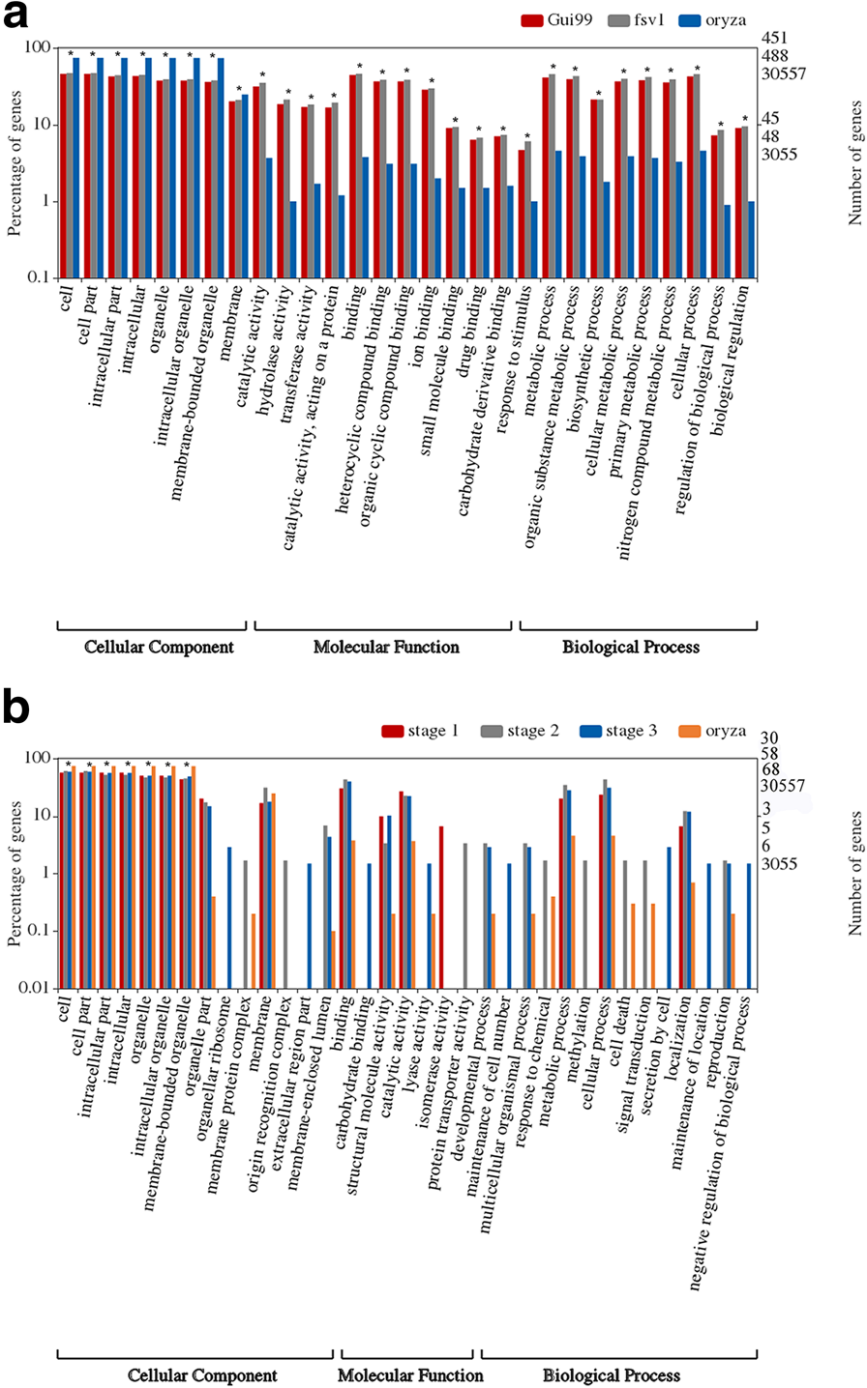
GO classifications of predicted target genes in different comparisons. In the figure, the predicted target genes are grouped to the secondary classification of hierarchical terms. **a** GO classification of the predicted target genes in different comparisons of Gui99 and *fsv1*. **b** GO classification of the predicted target genes between ovules of Gui99 and *fsv1* at stage 1, stage 2 and stage 3. “*” indicates significantly enriched GO terms, of which the *P*-value is below the significant level of 0.05. The figure just displays part GO terms and detailed information about the GO analysis is shown in Additional file 5: Table S5

To further understand the regulatory functions of lncRNAs, all predicted target genes were annotated to different GO function entries using Blast2GO (version 4.1.9) (https://www.blast2go.com/). With rice genome as a reference, the functional information of coherent target genes of differentially expressed lncRNAs at three developmental stages in *fsv1* and Gui99 ovules was classified using the WEGO website (http://wego.genomics.org.cn/). As shown in Additional file 5: Table S5, one lncRNA-related target gene could be annotated to different GO functional items, and a GO functional item could also be annotated with target genes of different lncRNAs. In the Gui99 and *fsv1* ovules, the coherent target genes of differentially expressed lncRNAs in A1 vs. A2, A2 vs. A3, and A1 vs. A3; and B1 vs. B2, B2 vs. B3, and B1 vs. B3 were classified into 188 and 192 GO terms, respectively (Additional file 6: Table S6). The most abundant GO terms in the cellular component, molecular function, and biological process categories were intracellular part (GO:0044424), organic cyclic compound binding (GO:0097159) and organic substance metabolic process (GO:0071704), respectively. In addition, the GO terms cell (GO:0005623), cell part (GO:0044464), intracellular part (GO:0044424), organelle (GO:0043226), membrane part (GO:0044425), and protein-containing complex (GO:0032991) in the cellular component category; hydrolase activity (GO:0016787), transferase activity (GO:0016740), heterocyclic compound binding (GO:1901363), and carbohydrate derivative binding (GO:0097367) in the molecular function category; and establishment of localization (GO:0051234), response to stimulus (GO:0050896), organic substance metabolic process (GO:0071704), cellular metabolic process (GO:0044237), and regulation of cellular process (GO:0050794) in the biological process category were significantly enriched in the three developmental stages of *fsv1* and Gui99 ovules (Fig. 7a). These GO terms were significantly enriched by the coherent target genes of differentially expressed lncRNAs, and they may participate significantly in the ovule developmental process.

To clarify the regulatory effects of lncRNAs on female gametophyte infertility, we further analyzed the functions of coherent target genes of differentially expressed lncRNAs in A1 vs. B1, A2 vs. B2, and A3 vs. B3 (Fig. 7). First, we can see that coherent target genes of differentially expressed lncRNAs in these three comparisons were annotated to several GO terms, such as cell (GO:0005623), cell part (GO:0044464), binding (GO:0005488), metabolic process (GO:0008152) and cellular process (GO:0009987). Second, there were some entries where the cellular component category was significantly enriched, including cell (GO:0005623), cell part (GO:0044464), intracellular part (GO:0044424), intracellular (GO:0005622), organelle (GO:0043226), intracellular organelle (GO:0043229) and membrane-bounded organelle (GO:0043227). Third, some coherent target genes were enriched into specific GO terms. For instance, only the comparison of A1 vs. B1 was enriched with target genes related to isomerase activity (GO:0016853). Additionally, only the comparison of A2 vs. B2 was enriched with genes associated with membrane protein complex (GO:0098796), origin recognition complex (GO:0000808), protein transporter activity (GO:0008565), response to chemical (GO:0042221), methylation (GO:0032259), and signal transduction (GO:0007165), and A3 vs. B3 was enriched in genes associated with organellar ribosome (GO:0000313), extracellular region part (GO:0044421), hormone metabolic process (GO:0042445), and maintenance of location (GO:0051235). In summary, these GO annotations indicate that the coherent target genes regulated by differentially expressed lncRNAs have different functions involved in various biological processes, and the lncRNAs might play important roles in female gametophyte sterility by regulating these coherent target genes.

### Prediction of lncRNAs acting as miRNA precursors

**Fig. 8:**
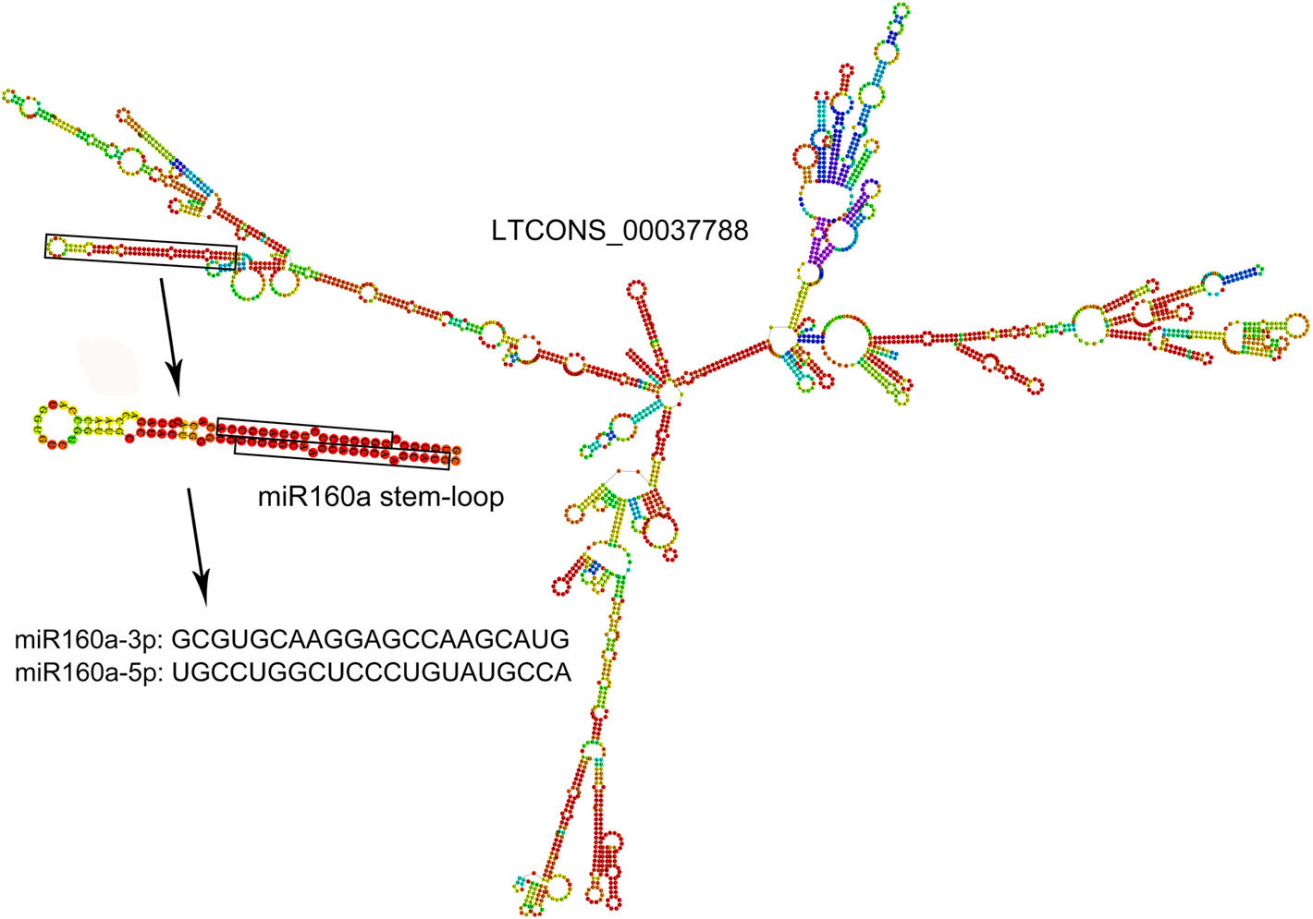
Prediction of miRNA Precursor of lncRNA (take LTCONS_00037788 for example)

lncRNAs can act as precursors for small RNA biosynthesis, so lncRNAs were aligned to miRBase to screen for miRNA precursors using BLAST. As a result, 53 expressed lncRNAs were identified as 44 miRNA precursors, of which 26 lncRNAs were significantly differentially expressed in *fsv1* and Gui99 ovules across three developmental stages (Table 2). As shown in Table 2, a lncRNA might serve as a miRNA precursor or several miRNA precursors, and several lncRNAs might also be precursors for the same miRNA. For example, LTCONS_00035558, LTCONS_00012871 and LTCONS_00040186 are the miRNA precursor of miR166d, miR172b and miR444b, respectively. LTCONS_00009363 is the miRNA precursor of osa-miR439a, osa-miR439b, osa-miR439c, osa-miR439d, osa-miR439e, osa-miR439f, osa-miR439g and osa-miR439h, and it was significantly downregulated in the comparisons of A1 vs. A3 and B1 vs. B3. In addition, LTCONS_00037942, LTCONS_00037941 and LTCONS_00037940 are miRNA precursors of osa-miR156d, of which LTCONS_00037942 was significantly upregulated in the comparison of A1 vs. A2, B1 vs. B3 and downregulated in A2 vs. A3, A2 vs. B2. To visualize the relationship between lncRNAs and miRNA precursors, the RNAfold web server (http://rna.tbi.univie.ac.at/cgi-bin/RNAWebSuite/RNAfold.cgi) was used to predict the secondary structures of several lncRNAs and miRNA precursors. For instance, the secondary structure prediction of LTCONS_00037788 shows that it has multiple stem-loop structures, one of which might be cleaved by an endonuclease to release the precursor sequence of osa-miR160a and eventually form mature osa-miR160a-3p and osa-miR160a-5p (Fig. 8). The expression level of LTCONS_00037788 consistently decreased over ovule development in *fsv1* and Gui99 (A1 vs. A2, A2 vs. A3, A1 vs. A3, B1 vs. B2, B2 vs. B3, B1 vs. B3), which may drive expression changes for miR160a and its target genes to affect ovule development and female gametophyte fertility.

**Table 2:** Prediction of miRNA precursor of lncRNA

MiRNA ID	LncRNA ID	MiRNA precursor length (nt)	LncRNA length (nt)	Alignment length (nt)	*E*-value	Alignment ratio
osa-miR1428b	LTCONS_00009984*	124	22,003	124	3.00E-65	1
osa-miR1428b	LTCONS_00009985*	124	20,702	124	3.00E-65	1
osa-miR1428b	LTCONS_00009986*	124	20,691	124	3.00E-65	1
osa-miR1428b	LTCONS_00009987*	124	22,838	124	3.00E-65	1
osa-miR1428d	LTCONS_00009984*	124	22,003	124	9.00E-44	0.919
osa-miR1428d	LTCONS_00009985*	124	20,702	124	9.00E-44	0.919
osa-miR1428d	LTCONS_00009986*	124	20,691	124	9.00E-44	0.919
osa-miR1428d	LTCONS_00009987*	124	22,838	124	9.00E-44	0.919
osa-miR156d	LTCONS_00037940	129	1438	129	3.00E-68	1
osa-miR156d	LTCONS_00037941	129	7353	129	3.00E-68	1
osa-miR156d	LTCONS_00037942*	129	5325	129	3.00E-68	1
osa-miR156i	LTCONS_00042575	90	1718	90	4.00E-45	1
osa-miR159b	LTCONS_00000263	188	2026	188	3.00E-103	1
osa-miR160a	LTCONS_00032617	88	6768	88	6.00E-44	1
osa-miR160a	LTCONS_00037788*	88	2442	88	6.00E-44	1
osa-miR160b	LTCONS_00074702	131	2815	131	8.00E-60	0.921
osa-miR162a	LTCONS_00035247*	171	2307	171	4.00E-93	1
osa-miR166b	LTCONS_00079180*	206	6730	206	9.00E-107	0.981
osa-miR166d	LTCONS_00035558*	125	3329	125	8.00E-66	1
osa-miR166h	LTCONS_00042628*	119	4024	119	3.00E-62	1
osa-miR166k	LTCONS_00042628*	127	4024	127	5.00E-67	1
osa-miR171c	LTCONS_00062303*	99	3985	99	8.00E-47	0.98
osa-miR172b	LTCONS_00012871*	238	4300	238	6.00E-133	1
osa-miR1846d	LTCONS_00006050	116	5861	116	2.00E-60	1
osa-miR1846d	LTCONS_00006052*	116	5547	116	2.00E-60	1
osa-miR1846d	LTCONS_00006054	116	4851	116	2.00E-60	1
osa-miR1848	LTCONS_00032626	63	4883	69	4.00E-07	0.921
osa-miR1850	LTCONS_00070604	133	1974	133	5.00E-67	0.985
osa-miR1863a	LTCONS_00033360*	374	8365	374	0	1
osa-miR1863a	LTCONS_00033362	374	12,189	374	0	1
osa-miR1863a	LTCONS_00033363	374	11,237	374	0	1
osa-miR1863a	LTCONS_00033364*	374	8172	374	0	1
osa-miR1863a	LTCONS_00033365*	374	10,187	374	0	1
osa-miR1863a	LTCONS_00033366	374	12,392	374	0	1
osa-miR1863a	LTCONS_00033368*	374	10,972	374	0	1
osa-miR1863a	LTCONS_00033369*	374	10,069	374	0	1
osa-miR1863a	LTCONS_00033371*	374	12,855	374	0	1
osa-miR1863b	LTCONS_00026174	360	10,196	360	4.00E-172	0.981
osa-miR1863c	LTCONS_00026174	291	10,196	291	1.00E-140	0.979
osa-miR1878	LTCONS_00096092	100	6930	100	2.00E-41	0.98
osa-miR2118j	LTCONS_00056168	164	5137	164	1.00E-74	0.936
osa-miR2118m	LTCONS_00056168	170	5137	170	3.00E-78	0.982
osa-miR2867	LTCONS_00020748	101	4991	100	3.00E-46	0.98
osa-miR2867	LTCONS_00020749	101	5318	100	3.00E-46	0.98
osa-miR390	LTCONS_00053270*	91	3035	91	4.00E-42	0.967
osa-miR394	LTCONS_00042593*	110	3523	110	6.00E-57	1
osa-miR399i	LTCONS_00038421	116	5367	116	2.00E-60	1
osa-miR439a	LTCONS_00009363*	93	5016	93	7.00E-47	1
osa-miR439b	LTCONS_00009363*	60	5016	58	2.00E-21	0.917
osa-miR439c	LTCONS_00009363*	93	5016	93	7.00E-47	1
osa-miR439d	LTCONS_00009363*	98	5016	98	1.00E-42	0.969
osa-miR439e	LTCONS_00009363*	98	5016	98	8.00E-50	1
osa-miR439f	LTCONS_00009363*	96	5016	96	1.00E-48	1
osa-miR439g	LTCONS_00009363*	88	5016	87	2.00E-43	0.989
osa-miR439h	LTCONS_00009363*	99	5016	99	5.00E-48	0.99
osa-miR444a	LTCONS_00089505	126	4370	126	2.00E-54	0.939
osa-miR444b	LTCONS_00040186*	138	2386	138	2.00E-73	1
osa-miR444d	LTCONS_00041518	143	2640	143	2.00E-76	1
osa-miR444d	LTCONS_00041519	143	2656	159	2.00E-54	1
osa-miR5150	LTCONS_00055710	79	5612	79	5.00E-29	0.975
osa-miR5179	LTCONS_00033804*	224	5064	224	1.00E-124	1
osa-miR5179	LTCONS_00033805*	224	5393	224	1.00E-124	1
osa-miR535	LTCONS_00023938	94	7835	94	7.00E-44	0.968
osa-miR6246	LTCONS_00043017	84	4204	84	5.00E-32	0.976
osa-miR7695	LTCONS_00001730	487	2728	487	0	1
osa-miR7695	LTCONS_00001731	487	2349	487	0	1
osa-miR812q	LTCONS_00006225*	242	3184	243	2.00E-83	0.917

*indicated that significantly differentially expressed lncRNAs

### lncRNAs act as decoys to compete with mRNAs for combining to miRNAs

**Fig. 9:**
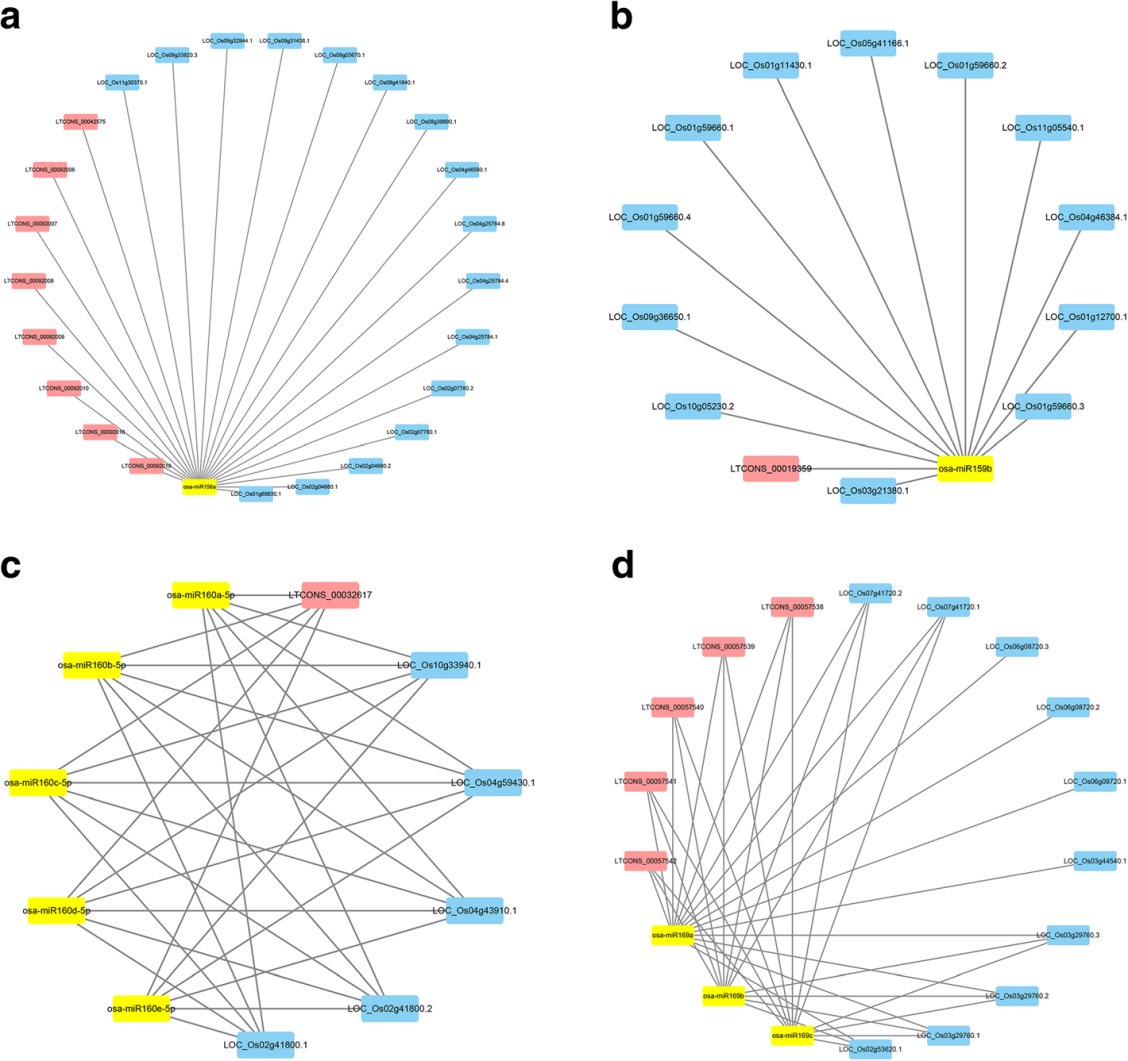
The network of lncRNAs, miRNAs and mRNAs

Studies have shown that lncRNAs can be used as decoys via pairing to miRNAs to prevent target mRNA binding, thus maintaining the stability of mRNA expression levels [11, 30]. To further understand the regulatory relationship between lncRNA, miRNA and mRNA during ovule development and female gametophyte abortion, a lncRNA-miRNA-mRNA expression interaction network was constructed in combination with the miRNA sequencing data performed by Yang et al. [20]. A total of 108 differentially expressed miRNAs were targeted by 114 lncRNAs and 330 mRNAs in the network (Additional file 7: Table S7), and Fig. 9 shows that most lncRNAs have a significantly weaker ability to compete with miRNA target sites than mRNAs. For example, miR156a had 24 target sites including 8 lncRNAs and 16 mRNAs, of which *OsSPL14* and *OsSPL16* were important transcription factor genes (Fig. 9a). The miR159b were targeted by LTCONS_00019359 and 12 mRNAs, including 6 transcripts derived from transcription factor genes *OsGAmyb*, *Osmyb5*, *OsDUO1* and a calmodulin gene *OsCML27* (Fig. 9b). We also found that different miRNAs simultaneously regulate several target sites; for example, the target sites of miR160a-5p, miR160b-5p, miR160c-5p, miR160d-5p, miR160e-5p are LTCONS_00032617, *OsARF8*, *OsARF10*, *OsARF13*, *OsARF22* (Fig. 9c). Moreover, the target genes of miR169a partly overlap with miR169b and miR169c, e.g., in LTCONS_00057538, LTCONS_00057539, LTCONS_00057540, *OsHAP2E*, and *OsHAP2G*, while other target genes were specific, such as *OsHAP2H* (Fig. 9d). In addition, the target genes of miR444b.1 and miR444c.1, LTCONS_00041518 and LTCONS_00041519 were competing with three MADS-box genes *OsMADS23*, *OsMADS27*, *OsMADS57* and several other genes.

### Validation of the sequencing data by qRT-PCR

In our study, the expression profiles of seven lncRNAs and their predicted differentially expressed target genes were randomly selected for verification by qRT-PCR analysis. After verification, the qRT-PCR expression patterns of lncRNAs and their target genes were consistent with the sequencing results, indicating that our sequencing results were accurate. The primers used for qRT-PCR are available in Additional file 1: Table S1 and the qRT-PCR results are shown in Additional file 8: Figure S1.

## Discussion

Recent studies have shown that lncRNAs play important roles in plant growth and sexual reproduction [13, 31]. In particular, lncRNAs have been reported to be involved in the male fertility of several plants [12, 32, 33], but the roles of lncRNAs in female fertility have not yet been elucidated. In our study, high-throughput sequencing techniques were used to examine the expression of lncRNAs in the ovules of a high-frequency female-sterile rice line and a wild-type rice line at different developmental stages. As a result, a total of 2965 lncRNAs across the entire genome were screened after sequencing and bioinformatics analysis. To confirm sequencing quality, we randomly selected six lncRNAs and their related target genes for qRT-PCR validation. The results showed that the expression levels of lncRNAs and mRNAs obtained by sequencing were in accordance with our qRT-PCR results, indicating that the sequencing data were reliable. Moreover, a total of 1131 lncRNAs revealed significant differential expression between *fsv1* and Gui99 ovules after differential expression analysis, suggesting that lncRNAs may be an important regulator involved in ovule development and female gametophyte abortion.

Rice ovule development is a very complex and delicate process involving multiple genes and diverse biological pathways [22]. Studies have shown that there is “cross-talk” between ovule sporophytic tissue and the female gametophyte; hence, abnormal expression of genes related to ovule development might affect the fertility of the female gametophyte [15, 34, 35]. As an important gene expression regulator, lncRNAs have been reported to be involved in ovule development and abortion of the female gametophyte in plants [14, 36]. In *Arabidopsis*, cytochrome P450 monooxygenase is involved in the initiation of `ty 5female gametogenesis and chromosome pairing during meiosis of MMC [37, 38]. In our study, LTCONS_00000849, LTCONS_00005124, LTCONS_00035676, LTCONS_00042496, LTCONS_00042583 and LTCONS_00078014 were significantly downregulated in the *fsv1* and Gui99 ovule developmental stages. All of these lncRNAs were predicted to target cytochrome P450 monooxygenase genes *CYP90D2* (LOC_Os01g10040) and *CYP734A6* (LOC_Os01g29150), and downregulation of these lncRNAs might result in changes in the expression levels of the cytochrome P450 monooxygenase genes that lead to the abnormal development of the ovule and female gametophyte. Cell cycle regulation is essential for normal plant development [39]. In *Arabidopsis*, *RETINOBLASTOMA-RELATED* (*RBR*) loss-of-function mutants lack the ability to transition from the G1 to S phase of the mitotic cell cycle, and the central cell of the mature female gametophyte becomes multinucleated, indicating that cell cycle arrest fails [40, 41]. However, the activation of the cyclin gene *CYCD7;1* in the central cell overcomes cell cycle arrest in the female gametophyte [42]. In the present study, two significantly downregulated lncRNAs in the *fsv1* and Gui99 ovules, LTCONS_00007790 and LTCONS_00005124, targeting two cyclin genes, *CycA1;1* (LOC_Os01g13260) and *CycB1;1* (LOC_Os01g59120), respectively, might affect the cell cycle and result in female gametophyte abortion.

Previous studies have shown that auxin, one of the most important plant hormones, plays an important role in the development of plant ovules and female gametophytes. Therefore, changes in the expression levels of auxin-related genes in ovules might influence the normal development of the female gametophyte [17, 43]. In this study, LTCONS_00037788 and LTCONS_00030633, which were significantly differentially expressed in *fsv1* and Gui99 ovules, were predicted to target two auxin response factor genes, *OsARF6* (LOC_Os02g06910) and *OsARF24* (LOC_Os12g29520), respectively. The differentially expressed lncRNAs may affect the female gametophyte by affecting the auxin response. As important transcriptional regulatory elements, transcription factors play crucial roles in the reproductive process of female plants [44–46]. In our data, LTCONS_00004164, LTCONS_00070807, LTCONS_00011471, and LTCONS_00042441 were significantly downregulated; LTCONS_00004164 and LTCONS_00070807 targeted two MYB transcription factor genes, *Osmyb4* (LOC_Os01g50110) and *Osmyb55* (LOC_Os05g48010), respectively, and LTCONS_00011471 and LTCONS_00042441 targeted the two bZIP transcription factor genes *OsbZIP09* (LOC_Os01g59760) and *OsbZIP24* (LOC_Os02g58670), respectively. Previous studies have shown that MYB transcription factors can regulate female reproduction in flowering plants and that they are required for cellularization and differentiation during female gametogenesis, while the loss of MYB transcription factors reduces the number of ovules to cause female fertility reduction [45, 47]. The bZIP transcription factor genes were reported to be expressed preferentially in ovules and might regulate genes during cotton fiber elongation in *Gossypium hirsutum*, and a bZIP protein participates in the determination of floral organ number in *Arabidopsis thaliana* [48, 49]. Therefore, the significantly differential expression of these lncRNAs might affect the expression of transcription factor genes and lead to female gametophyte abortion.

As important gene regulators, miRNAs are widely involved in ovule development [50, 51]. changes to the expression levels of lncRNAs that act as miRNA precursors might generate important effects on miRNA expression levels [52, 53]. In tomatoes, miR156 is expressed in ovarian tissue, including the placenta and ovules, and mediates the cleavage of SPB-box genes that are involved in gynoecia development [54]. Furthermore, the detection of miRNA expression patterns in *fsv1* and Gui99 ovules at the three developmental stages also revealed that miR1561-5p is significantly differentially expressed during stage 1, suggesting that miR156 has an important effect on female gametophyte abortion [20]. In this study, three lncRNAs, LTCONS_00037940, LTCONS_00037941 and LTCONS_00037942, were determined to be the precursors of miR156d, and LTCONS_00042575 was determined to be a precursor of miR156i. LTCONS_00037942 was upregulated in stage 3 of the Gui99 and *fsv1* ovules. Changes in the expression of these precursor lncRNAs might cause alterations to miR156 function and thus affect its interaction with target genes, leading to abortion of the female gametophyte. As mentioned above, auxin is important for normal ovule and female gametophyte development. In Arabidopsis, miR160 targets *ARF10*, *ARF16* and *ARF17*, and abnormal expression causes multiple developmental reproductive defects, such as premature inflorescence development, reduced petal size, and infertility [55]. In our study, two lncRNAs, LTCONS_00032617 and LTCONS_00037788, were identified as precursors of miR160a, and LTCONS_00074702 was found to be a precursor of miR160b. Among them, the significant downregulation of LTCONS_00037788 lead to a continuous decrease of miR160a expression in the development of *fsv1* ovules [20]. The target genes of miR160a (such as *OsARF8, OsARF10, OsARF18, OsARF22*) were also significantly differentially expressed, indicating a potential role for miR160a in the regulation of ovule development and fertile female gametophyte formation [20]. Moreover, three significantly downregulated lncRNAs, LTCONS_00079180, LTCONS_00042628 and LTCONS_00042628, were precursors of miR166b, miR166h and miR166k, respectively, and their expression changes lead to the downregulation of miR166b, miR166h and miR166k in *fsv1* ovule development [20]. Thus, downregulation of miR166 expression levels might be relevant to fertile female gametophyte formation, as previous studies have found that miR166 can regulate HD-ZIP III family genes which are involved in female gametophyte formation [56, 57]. In rice, overexpression of miR172 delayed the transition from spikelet meristem to flower meristem, leading to floral developmental defects and lower fertility [58]. LTCONS_00012871, the precursor of miR172b, was upregulated at the mature female gametophyte stage in both Gui99 and *fsv1* ovules [20]. The significant increase in expression may be one of the causes of *fsv1* female gametophyte abortion. In addition, LTCONS_00089505, LTCONS_00040186, LTCONS_00041518, LTCONS_00041519, LTCONS_00033804 and LTCONS_00033805 were the precursors of miR444a/b/d and miR5179 in *fsv1* and Gui99 ovules. MiR444 and miR5179 have been shown to target MADS-box genes [59, 60], which are crucial for floral organs and are differentially expressed in *fsv1* and Gui99 ovules [20]. These lncRNAs may contribute to the development of the female gametophyte.

One of the important functions of lncRNA is its role as a decoy molecule, and studies have shown that lncRNAs can regulate gene expression by binding to miRNAs in competition with mRNAs [11, 30]. We discussed miR156 above, and we found two target genes of miR156a/b-5p/c-5p/d/e/f-5p/g-5p/h-5p/i/j-5p/l-5p, *OsSPL14* and *OsSPL16* that might be involved in female gametophyte abortion [19]. Additionally, six significantly differentially expressed lncRNAs, LTCONS_00092006, LTCONS_00092007, LTCONS_00092008, LTCONS_00092010, LTCONS_00092016 and LTCONS_00092018, were targeted by these miRNAs, indicating that the lncRNAs may compete with *OsSPL14* and *OsSPL16* to affect their expression levels and influence female gametophyte fertility. As described above, auxins play an essential role in the formation of rice ovules and female gametophytes. In our study, we found that miR160a/b/c/d/e-5p targets four uniform ARF genes, *OsARF8*, *OsARF10*, *OsARF13* and *OsARF22*. As the other target gene of miR160a/b/c/d/e-5p, LTCONS_00032617 may participate in normal ovule and female gametophyte development by affecting auxin gene expression through competitive miRNA binding. In addition, LTCONS_00041518 competes with three MADS-box genes, *OsMADS23*, *OsMADS27* and *OsMADS57,* for binding to miR444b.1 and miR444c.1. This may also be an important factor affecting the fertility of the female gametophyte.

## Conclusions

In summary, we examined the ovular expression patterns of lncRNAs at different developmental stages and identified and screened lncRNAs in the *fsv1* high-frequency female-sterile rice line and the wild-type rice line Gui99. In our study, functional modes were revealed for lncRNAs, including regulation of coherent target genes, acting as precursors of miRNAs, and competing with mRNAs for binding to miRNAs. Functional analysis of coherent target genes uncovered that lncRNAs are involved in multiple biological processes, such as signal transduction and hormone metabolism. We hypothesize that abortion of a female gametophyte is a complex and delicate biological event, and changes in lncRNAs are important factors that affect this event. In conclusion, the studies above reveal whole genome expression profiles of lncRNAs in *fsv1* high-frequency female-sterile and Gui99 wild-type rice ovules, providing important evidence for further research on the molecular mechanisms of female gametophyte fertility.

## Additional files

Additional file 1: **Table S1.** The primers used for quantitative real-time PCR. (XLSX 11 kb)

Additional file 2: **Table S2.** Summary of reads mapping to rice genome in three developmental stages ovules of *fsv1* and Gui99. (XLSX 10 kb)

Additional file 3: **Table S3.** The normalized FPKM values for all lncRNAs and mRNAs in all samples. (XLSX 2100 kb)

Additional file 4: **Table S4.** Summary of significantly differentially expressed lncRNA between ovules of two rice lines at three developmental stages. (XLSX 163 kb)

Additional file 5: **Table S5.** The coherent target genes of significantly differentially expressed lncRNAs in all comparisons (GO annotation of these genes were listed). (XLSX 180 kb)

Additional file 6: **Table S6.** The GO terms which were enriched by coherent target genes of differentially expressed lncRNAs in different comparisons. (XLSX 21 kb)

Additional file 7: **Table S7.** The network of lncRNAs, miRNAs and mRNAs. (XLSX 35 kb)

Additional file 8: **Figure S1.** The RNA-seq data and qRT-PCR validation of seven lncRNAs and their target protein-coding genes. The relative expression levels of seven lncRNAs and their target protein-coding genes were shown. A lncRNA can have one or more target protein-coding genes. The bars denote the standard deviation. (PDF 746 kb)

## Abbreviations

CNCI: Coding Non Coding Index
CPC: Coding Potential Calculator
FDR: False discovery rate
FLC: FLOWER LOCUS C
FPKM: Fragments per kilobase of transcript per million mapped reads
GO: Gene Ontology
incRNA: Intronic non-coding RNA
LDMAR: Long-day-specific male-fertility-associated RNA
lincRNA: Intergenic non-coding RNA
lncRNA: Long non-coding RNA
MMC: Megaspore mother cell
NAT: Antisense transcript
qRT-PCR: quantitative real-time PCR
ssRNA-seq: Transcriptome strand-specific RNA sequencing
