# Supplementary figures and images for: Identification of lncRNAs involved in rice ovule development and female gametophyte abortion by genome-wide screening and functional analysis
Source: BMC Genomics. 2019 Jan 28;20:90. doi: 10.1186/s12864-019-5442-6 (PMC6348626; doi:10.1186/s12864-019-5442-6)

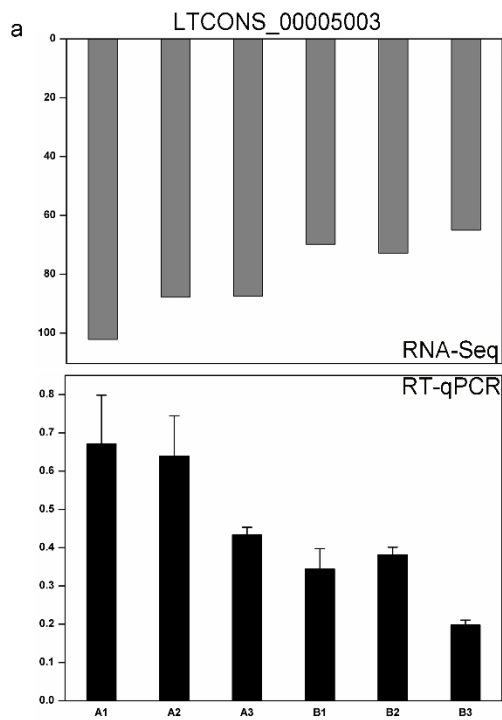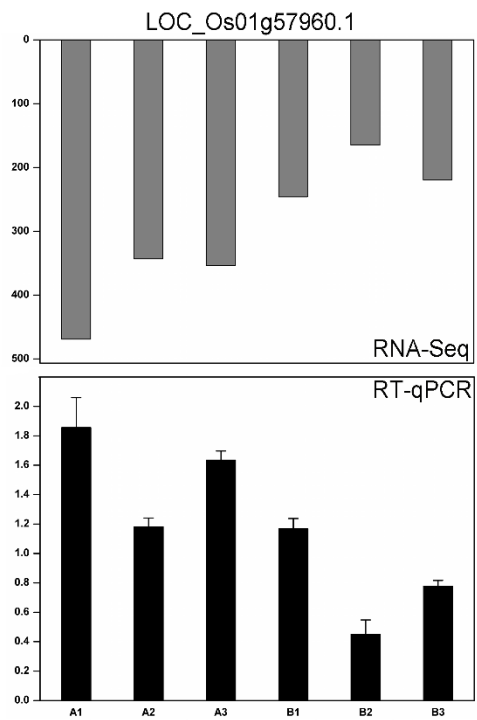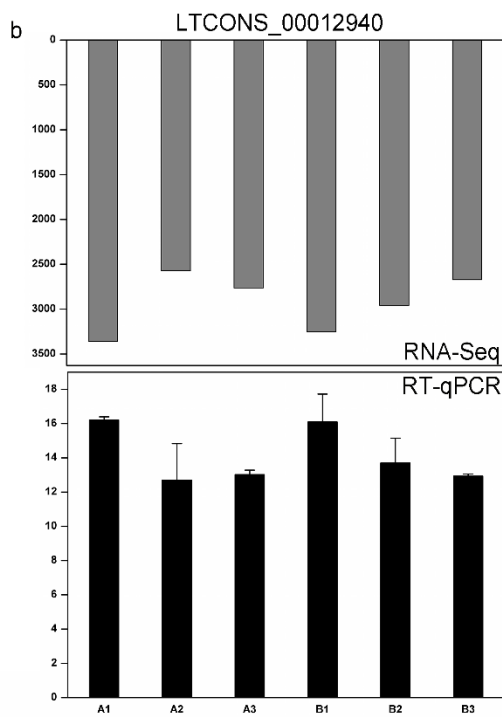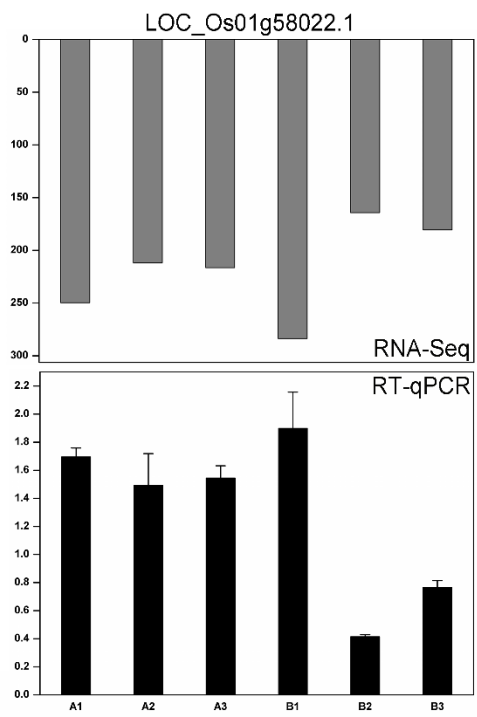

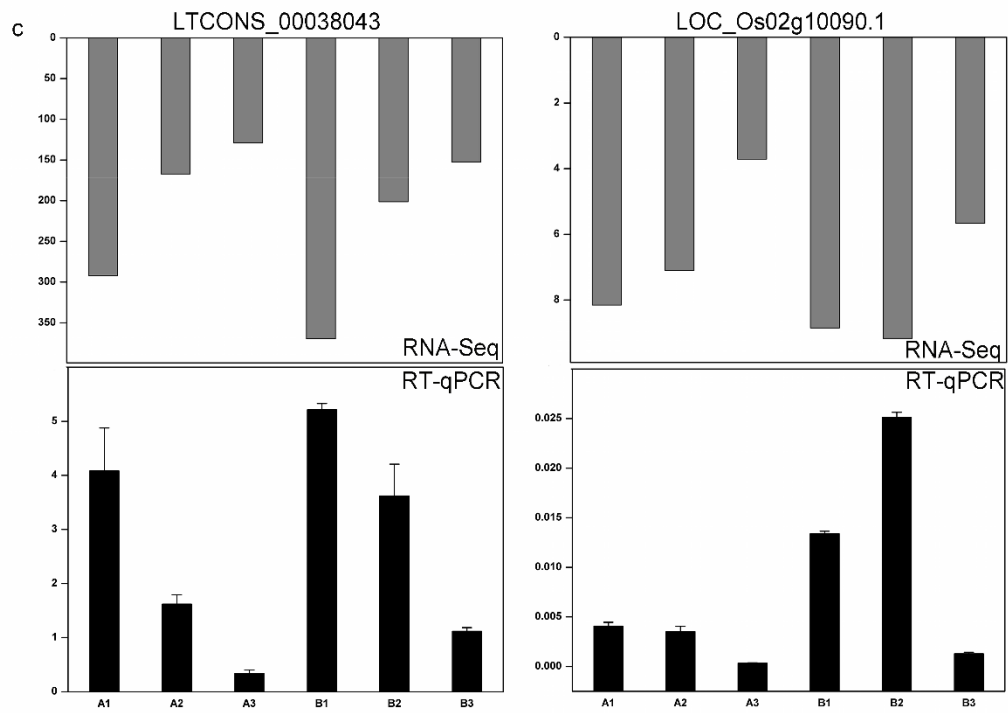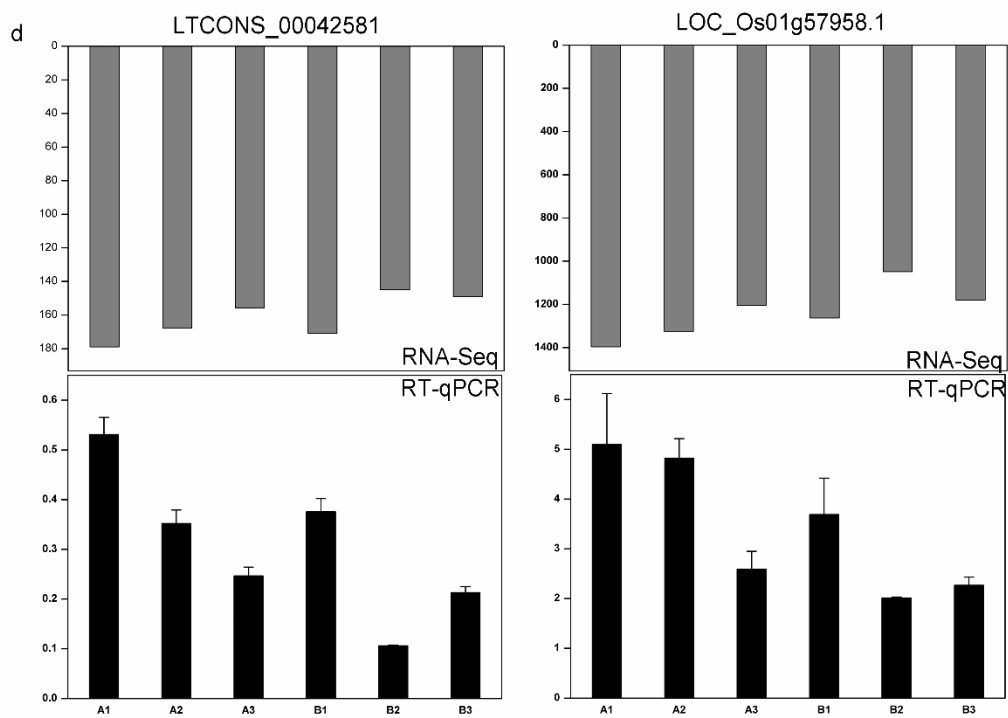

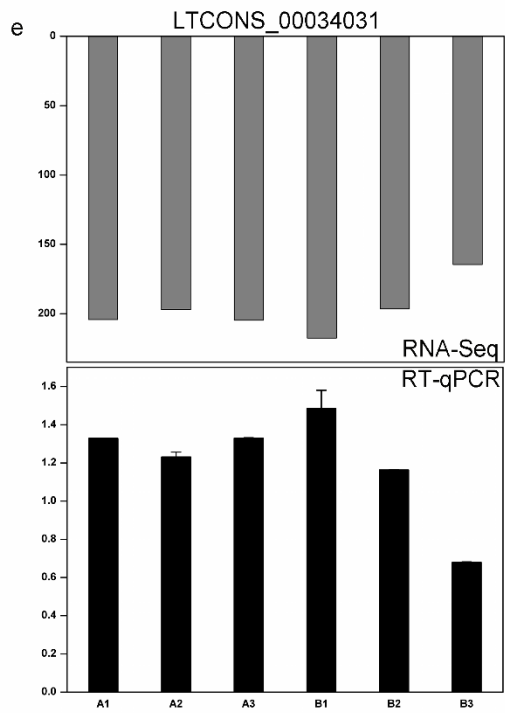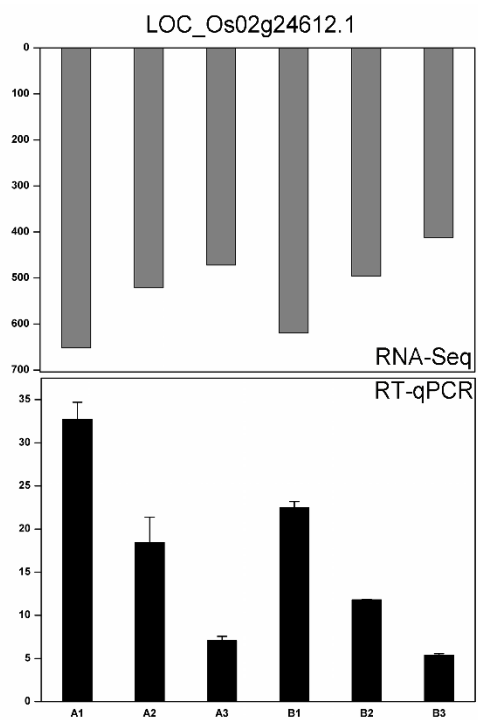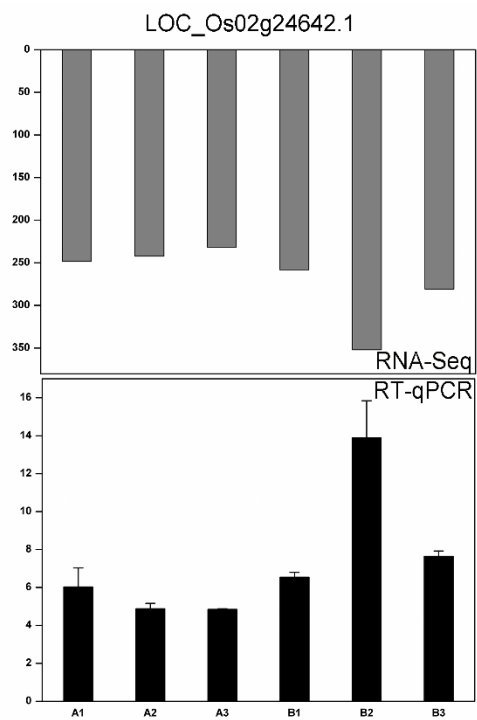

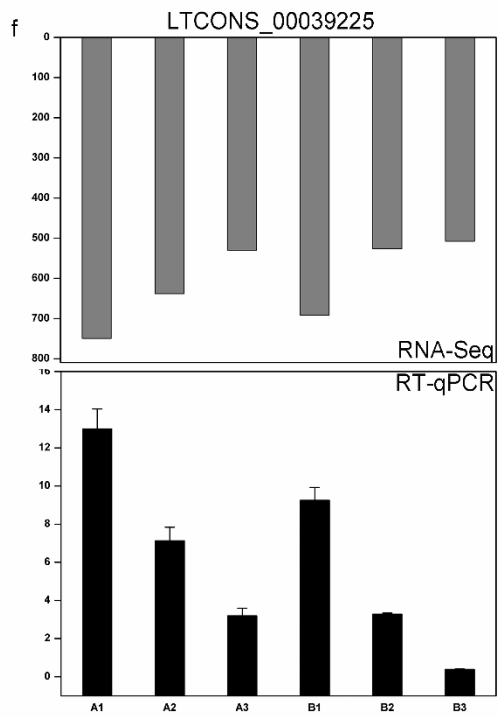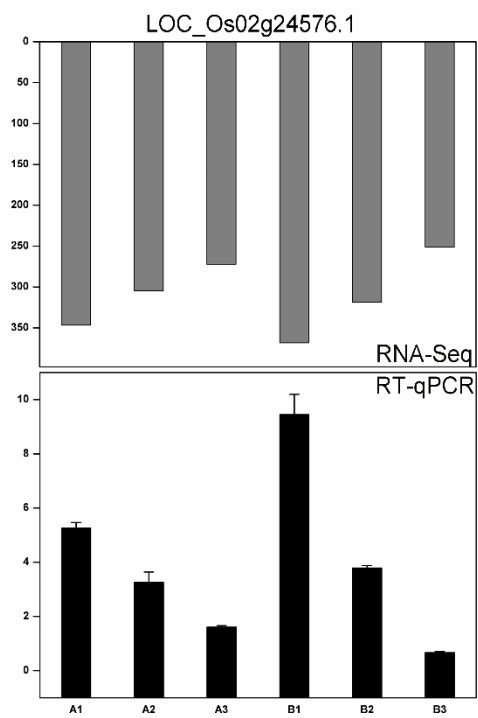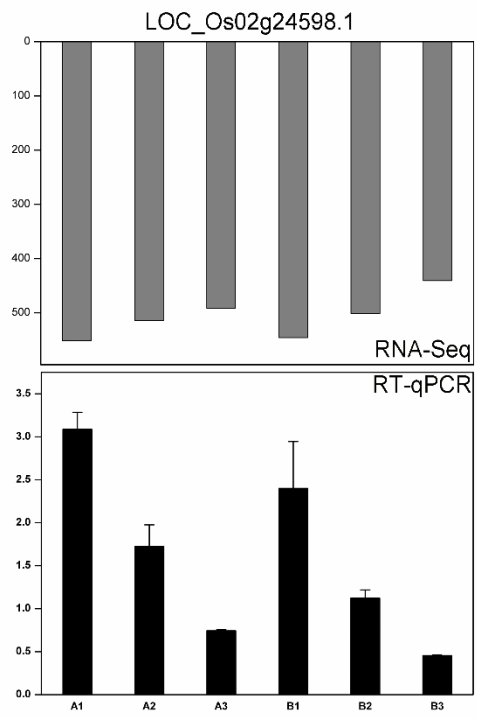

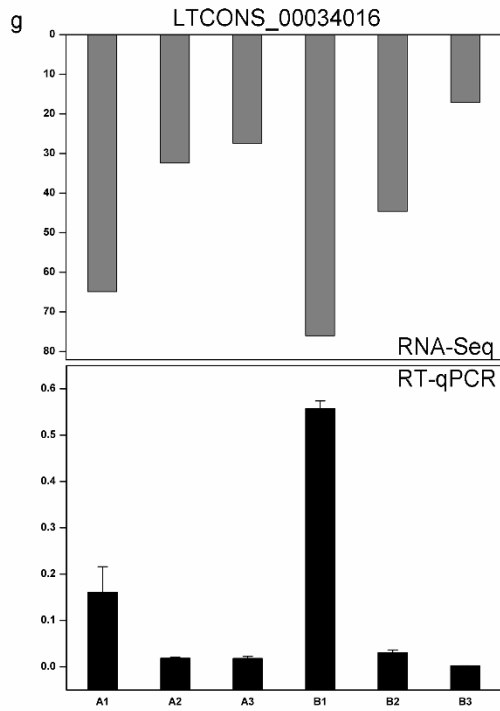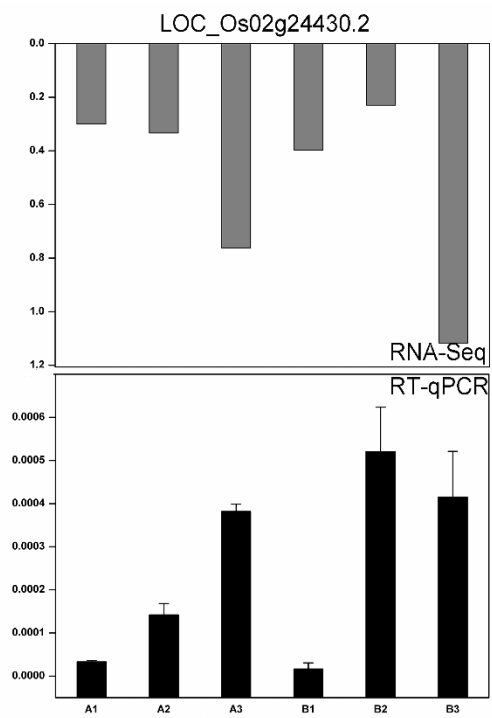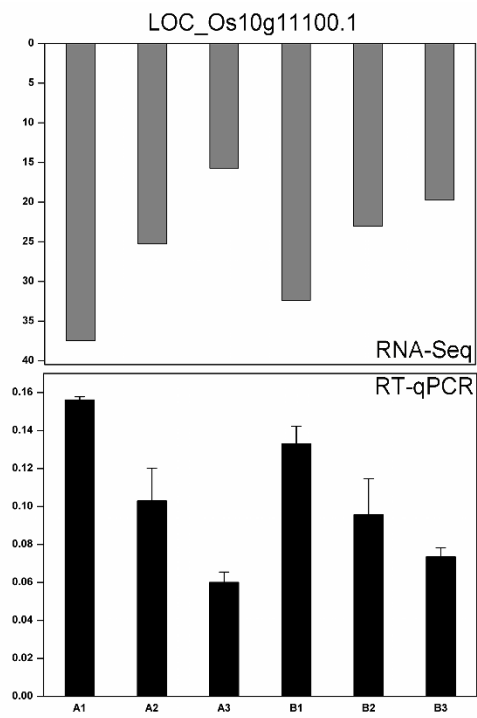

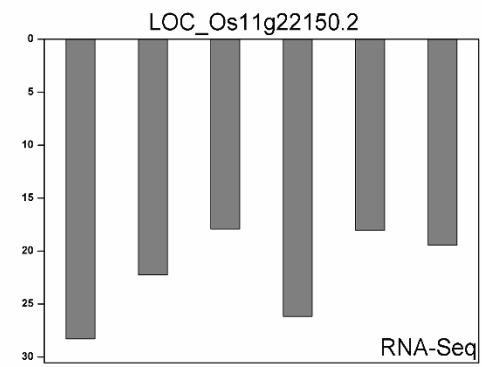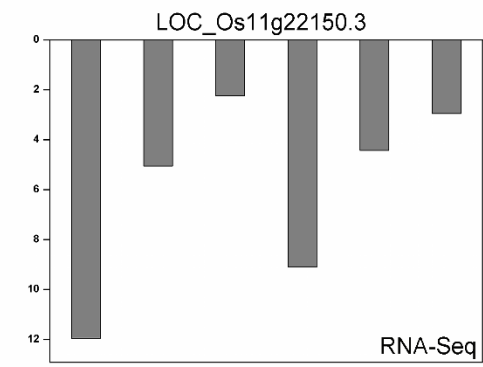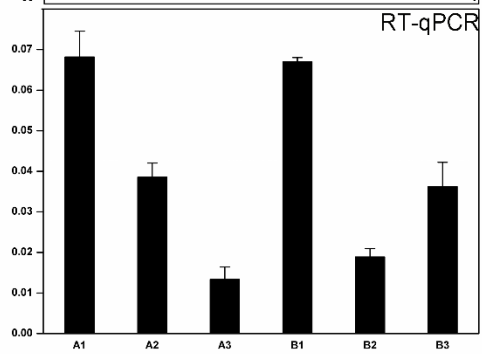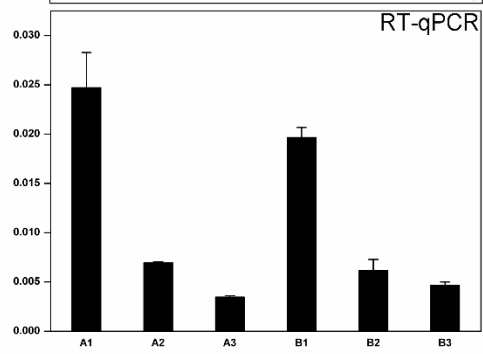

Supplement: Supplementary file 8 — Figure S1. The RNA-seq data and qRT-PCR validation of seven lncRNAs and their target protein-coding genes. The relative expression levels of seven lncRNAs and their target protein-coding genes were shown. A lncRNA can have one or more target protein-coding genes. The bars denote the standard deviation. (PDF 746 kb) [file 12864_2019_5442_MOESM8_ESM.pdf]
